# Reporting studies on time to diagnosis: proposal of a guideline by an international panel (REST)

**DOI:** 10.1186/s12916-016-0690-7

**Published:** 2016-09-27

**Authors:** Elise Launay, Jérémie F. Cohen, Patrick M. Bossuyt, Pierre Buekens, Jonathan Deeks, Timothy Dye, Richard Feltbower, Andrea Ferrari, Michael Kramer, Mariska Leeflang, David Moher, Karel G. Moons, Erik von Elm, Philippe Ravaud, Martin Chalumeau

**Affiliations:** 1 Obstetrical, Perinatal and Pediatric Epidemiology Research Team (Epopé), Center for Epidemiology and Statistics Sorbonne Paris Cité (CRESS), Paris Descartes University, INSERM U1153, Maternité de Port-Royal, 53 Avenue de l’Observatoire, 75014 Paris, France; 2 CHU de Nantes, Hôpital Mère-Enfant, Services de Pédiatrie Générale et d’Urgences Pédiatriques, Nantes, France; 3 Service de Pédiatrie Générale, Hôpital Necker-Enfants Malades; AP-HP; Université Paris Descartes, Paris, France; 4 Department of Clinical Epidemiology, Biostatistics and Bioinformatics, Academic Medical Center, University of Amsterdam, Amsterdam, The Netherlands; 5 School of Public Health and Tropical Medicine, Tulane University, New Orleans, LA USA; 6 Institute of Applied Health Research, University of Birmingham, Birmingham, UK; 7 Biomedical Informatics, Clinical and Translational Science Institute, University of Rochester, Rochester, NY USA; 8 Division of Epidemiology and Biostatistics, School of Medicine, University of Leeds, Leeds, LS2 9JT UK; 9 Pediatric Oncology Unit, Fondazione IRCCS Istituto Nazionale Tumori, Milano, Italy; 10 Departments of Pediatrics and of Epidemiology, Biostatistics and Occupational Health, McGill University Faculty of Medicine, Montreal, Quebec Canada; 11 Centre for Practice Changing Research, Ottawa Hospital Research Institute, School of Epidemiology, Public Health and Preventive Medicine, University of Ottawa, Ottawa, ON Canada; 12 Julius Center for Health Sciences and Primary Care, UMC Utrecht, Utrecht, The Netherlands; 13 Cochrane Switzerland, Institute of Social and Preventive Medicine, Lausanne University Hospital, Lausanne, Switzerland; 14 Inserm UMR 1153, METHODS Team, Center for Epidemiology and Statistics Sorbonne Paris Cité (CRESS), Paris Descartes University, AP-HP, Paris, France

**Keywords:** Time to diagnosis, Reporting guideline, Risk of bias, Generalizability, Research methodology

## Abstract

**Background:**

Studies on time to diagnosis are an increasing field of clinical research that may help to plan corrective actions and identify inequities in access to healthcare. Specific features of time to diagnosis studies, such as how participants were selected and how time to diagnosis was defined and measured, are poorly reported. The present study aims to derive a reporting guideline for studies on time to diagnosis.

**Methods:**

Each item of a list previously used to evaluate the completeness of reporting of studies on time to diagnosis was independently evaluated by a core panel of international experts (n = 11) for relevance and readability before an open electronic discussion allowed consensus to be reached on a refined list. The list was then submitted with an explanatory document to first, last and/or corresponding authors (n = 98) of published systematic reviews on time to diagnosis (n = 45) for relevance and readability, and finally approved by the core expert panel.

**Results:**

The refined reporting guideline consists of a 19-item checklist: six items are about the process of participant selection (with a suggested flowchart), six about the definition and measurement of time to diagnosis, and three about optional analyses of associations between time to diagnosis and participant characteristics and health outcomes. Of 24 responding authors of systematic reviews, more than 21 (≥88 %) rated the items as relevant, and more than 17 (≥70 %) as readable; 19 of 22 (86 %) authors stated that they would potentially use the reporting guideline in the future.

**Conclusions:**

We propose a reporting guideline (REST) that could help authors, reviewers, and editors of time to diagnosis study reports to improve the completeness and the accuracy of their reporting.

**Electronic supplementary material:**

The online version of this article (doi:10.1186/s12916-016-0690-7) contains supplementary material, which is available to authorized users.

## Background

Time to diagnosis (TTD) is the interval from first alert symptoms to the diagnosis of a disease in a patient [1–3]. Studies on TTD may aim at (1) measuring the length of TTD and its evolution over time, (2) identifying the determinants of long TTD (i.e., relationship between TTD and patient or healthcare system characteristics), and/or (3) evaluating relationships between long versus short TTD and patient outcomes [1, 4]. The number of published studies on TTD is rapidly increasing [4]. TTD is also of increasing concern among patient advocacy groups, and some, such as the International Confederation of Childhood Cancer Parent Organizations, have established a reduction in TTD as a priority [5]. There are strong and common beliefs that longer TTD is associated with worse health outcomes, as revealed by various systematic reviews of the literature [1–3]. Furthermore, in PubMed, the definition of the Medical Subject Heading “early diagnosis” includes the following statement: ‘*Generally, early diagnosis improves prognosis and treatment outcome*’ [6]. However, many primary studies and systematic reviews do not report worse patient outcomes as a consequence of longer versus shorter TTD, or have found inverse relationships between longer TTD and better patient outcomes [1, 2]. These paradoxical associations are due to incomplete adjustment and residual confounding, as observed in studies of TTD of pediatric brain and bone tumors [2, 7, 8]. Another issue associated with “early diagnosis” is related to the risk of over-diagnosis and over-treatment [9].

Studies on TTD are most commonly based on series of diagnosed cases and therefore have specific design features exposing them to risk of bias and threats to the generalizability of their findings [1]. For example, bias may be introduced by how participants were selected, how TTD was defined and measured, and how the association with participant characteristics and health outcomes was assessed [1, 2, 4]. In a systematic review evaluating the quality of reporting of 50 studies on TTD, we found that these critical methodological aspects were rarely reported. Hence, critical appraisal of these studies by authors of systematic reviews was found frequently inadequate [4].

In a previous work, we showed that the reporting of primary studies on TTD was poor, notably for their specific design features. We therefore hypothesized that available reporting guidelines (e.g., STROBE [10], STARD [11], and Aarhus statement [12]) could be insufficient for accurate and complete reporting of studies on TTD [1, 4]. A first checklist was previously developed by some of us to evaluate the completeness of reporting of studies on TTD in the field of pediatrics [1]. The aim of the present study was to derive a Reporting guideline for studies on TTD, using this previous checklist and the expertise of two independent international panels [13].

## Methods

### General methodology

**Fig. 1:**
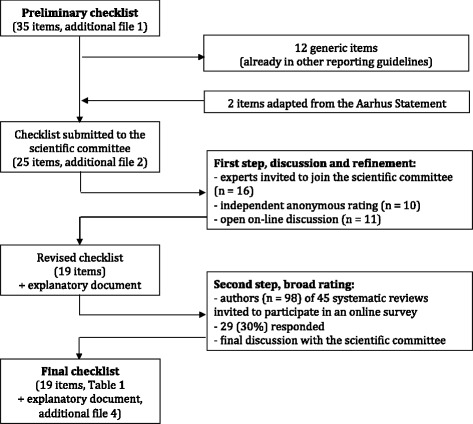
Flow of the refinement, rating, and final approval process of the checklist for the reporting of studies on time to diagnosis

We followed the EQUATOR network recommendations for developing reporting guidelines [13]. We used a three-step process: a discussion and refinement step with a core panel of experts (referred below as the “scientific committee”), a rating step in which we invited authors of all systematic reviews on TTD, and a final discussion and approval by the scientific committee (Fig. 1). We declared the development of the present reporting guideline to the EQUATOR network; a summary of our protocol was published online [14].

### First version of the checklist

A preliminary checklist of 25 items was submitted to the scientific committee. This checklist included 23 (of 35) items generated in previous work in which we had evaluated the quality of reporting of studies on TTD in the field of pediatrics (Additional file 1) [1]. These 23 items had been considered potentially related to risk of bias and threats to generalizability [1]. The 12 other items were generic (e.g., aim of the study and inclusion criteria) common with other related reporting guidelines (e.g., STROBE, STARD); they were not initially submitted to the scientific committee but were reintegrated during the discussion process (Fig. 1). Two items from the Aarhus statement concerning TTD definition and measurement were added [12].

### Discussion and refinement step

We identified 16 potential members for the scientific committee, comprising clinical research methodologists, clinical epidemiologists, clinicians, specialists in the field of TTD studies, and medical editors. We invited them to participate in an online survey by rating the relevance and readability of each item. We asked them to answer “yes” or “no” to the two following questions: “Is this item relevant?” and “Is this item readable?” Experts from the scientific committee were also invited to provide comments and to suggest additional items. After the first round of rating, we collated and anonymized all answers and comments, and sent them back to members of the scientific committee, together with a revised version of the checklist. Members of the scientific committee were then invited to provide feedback on the revised checklist and to propose modifications through an online general discussion. Revisions were made until we obtained agreement from all members of the scientific committee. The revised checklist was then used in the second broad rating step.

### Broad rating step

We invited the first author, last author and corresponding (if different) author of each systematic review on TTD identified in a previous methodological review [4] to participate in an online survey in SurveyMonkey®. Authors from this broad panel were asked to rate the relevance and the readability of each item using the same questions as in the core refinement step. We added the possibility to answer “I don’t know” to the question on relevance. Experts could also leave comments on each item or the checklist and were asked if they would use this reporting guideline to report a study on TTD in the future. To facilitate understanding of items specific to studies on TTD, we provided a document with explanations and examples alongside the survey. Non-responders were sent two reminders. None of the 11 experts in the scientific committee participated in the broad rating step.

### Finalization of the checklist and the explanatory document

Members of the scientific committee were invited to discuss deletion from the checklist for items with less than 50 % of agreement on relevance in the broad rating step, and to consider rephrasing for items with less than 50 % of agreement on readability. Comments of the broad rating panel were also used to complete the explanatory document. A final version of the reporting guideline, comprising a checklist and an explanatory document, was approved by the scientific committee through online discussions.

## Results

### Discussion and refinement step

**Fig. 2:**
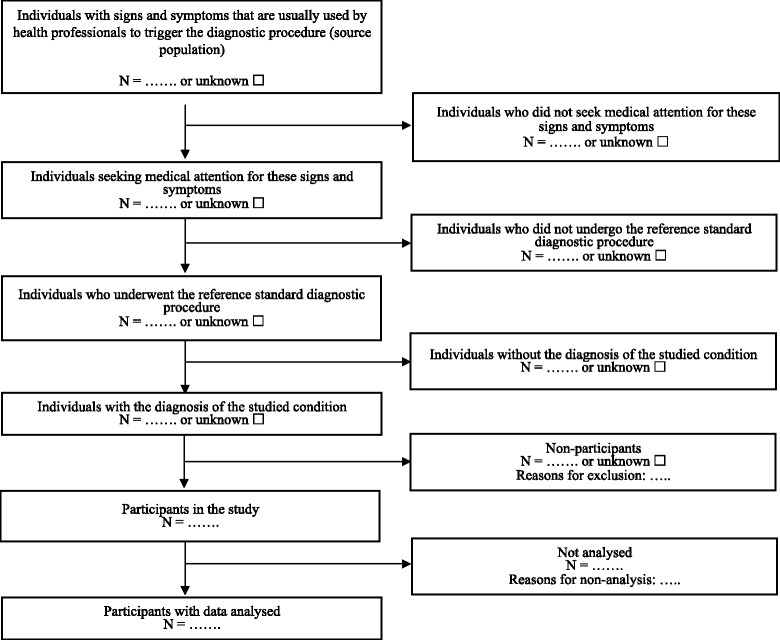
Template flowchart for studies on time to diagnosis (see item 14 on Table 1)

Among the 16 experts invited to form the scientific committee, 11 responded (PBo, PBu, JD, TD, RF, AF, MK, ML, DM, KM, EvE). Twenty of the 25 items were rated as relevant for the reporting of studies on TTD by seven or more experts (including 13 rated relevant by nine or more experts), three items were deleted because less than five experts rated them as relevant (Additional file 2), and 14 items were merged into six new items; other six generic items and a template for a flowchart (Fig. 2) were added. Nineteen items were rated readable by seven or more experts, with several comments suggesting rephrasing, and all items were partially or completely rephrased. After an online general discussion and approval by the scientific committee, we obtained consensus on a revised checklist consisting of 19 items.

### Broad rating step

Among 98 authors of 45 systematic reviews on TTD who were invited to participate, 29 (30 %) answered at least one question and 24 (24 %) rated all the 19 items. The topics of the systematic reviews written (or co-written) by the 98 authors were similar to those of the 29 responding authors (Additional file 3). Seventeen (59 %) of the 29 responding authors had already published a primary study on TTD, in addition to one or more systematic reviews.

All items were rated as relevant by at least 21 authors (88 % of the 24 responding authors). All items were rated as readable by at least 17 (71 %) of the 24 responding authors who rated all the items, while 19 items were rated as readable by at least 19 (80 % of the 24 responding authors) (Additional file 4). Among the 22 authors who answered the question, 19 (86 %) stated that they would potentially use the reporting guideline in the future. Among these, 10 had already conducted one or more primary studies on TTD. Three authors of systematic reviews commented that the Aarhus statement would be more appropriate to their fields of research.

### Final version of the checklist

Given that the 19 proposed items were deemed relevant and readable by a majority of respondents of the broad rating panel, we decided to submit an unchanged checklist to the scientific committee. The explanatory document was completed and also submitted to the scientific committee.

Both documents were endorsed by the scientific committee (Table 1 and Additional file 5). Six items of the checklist focus on the reporting of how participants were selected (see items 3–6 and 14–15 in Table 1) with a suggested flowchart to report each step of the participant selection process, from the source population (population with signs and symptoms that are usually used by health professionals to trigger the diagnostic procedure) to participants with data analyzed. Six items concern the reporting of the definition and measurement of TTD (items 7–10 and 16–17), and three items focus on the reporting of analyses of the association between TTD and participant characteristics and health outcomes (items 10, 12, and 17). Items 1–5, 7–9, 11, 13–16, and 18 are mandatory for all types of studies on TTD and other items are optional depending on the studied condition (item 6) or the aim of the study (items 10, 12, 17, and 19).

**Table 1:** Checklist for reporting studies on time to diagnosis (REST). More detailed explanation on items and examples are given in Additional file 5

Number	Items	Page^c^
Title		
1	Identify the article as a study on time to diagnosis	
Introduction		
2a^a^	Explain the scientific background and rationale for the study	
2b^a^	State specific objective(s)	
Methods		
3^a^	Describe the setting, location(s), and relevant dates, including periods of recruitment	
4	State eligibility criteria of participants (i.e., inclusion and exclusion criteria, especially diagnostic criteria)	
5	Describe the source population (i.e., the population with signs and symptoms that usually trigger healthcare professionals to initiate the diagnostic procedures) and how the participants were identified within it	
6^b^	State how known subgroups of participants with an inherent individual risk of short or long time to diagnosis were handled (e.g., by subgroup analysis, exclusion)	
7	Define time points (e.g., time of first signs and symptoms, time of diagnosis) and time intervals^b^ (e.g., patient or physician intervals)	
8^a^	State the methods used to collect study data (e.g., extraction from medical records, participant interview or questionnaires, analysis of an already existing database, other)	
9	Describe how time points were assessed (e.g., number of assessors, their qualifications)	
10a^b^	If the study aimed to evaluate associations between participant characteristics and time to diagnosis, state whether assessors of time to diagnosis were blinded to these characteristics	
10b^b^	If the study aimed to evaluate associations between time to diagnosis and participant health outcomes (e.g., survival), state whether assessors of time to diagnosis were blinded to these outcomes	
11	Describe the statistical methods used, including whether time to diagnosis was analyzed as a continuous or categorized variable (e.g., delayed versus not delayed)	
12^b^	If the study aimed to evaluate associations between time to diagnosis and other factors (e.g., participant characteristics or health outcomes), describe which confounders were considered and how they were chosen, measured and analyzed	
13^a^	Give a rationale for the sample size	
Results		
14	Report the number of individuals at each step of the selection process between the source population and participants with data analyzed and provide a flowchart (see example); give reasons for non-participation at each stage	
15^a^	Report demographic and clinical characteristics of participants	
16	Report the distribution of time to diagnosis	
17^b^	If associations between time to diagnosis and other factors (e.g., participant characteristics or health outcomes) were described, report measures of association and their precision (e.g., confidence intervals)	
Discussion		
18^a^	Summarize key results with reference to study objectives and discuss their potential clinical implications	
19a	Discuss sources of potential bias, including bias due to the selection of participants from the source population (e.g., undiagnosed cases) and to the assessment of time points	
19b^b^	If association between time to diagnosis and survival was studied, discuss possible lead-time bias	

^a^Items common with other reporting guidelines (CONSORT, STARD, STROBE) in their meaning

^b^Optional items depending on the studied condition or the study objectives

^c^Authors should precisely state the page number on which the item is reported, or NA if not applicable

## Discussion

We propose a new guideline (REST) for TTD studies to help authors, reviewers, and editors improve the reporting of these studies. The 19-item checklist (Table 1), along with the explanatory document (Additional file 5), have been approved by an international panel of experts in the field of research methodology, epidemiology and TTD studies, journal editors, and also by 71 to 88 % of responding authors of systematic reviews on TTD published before 2014. Our reporting guideline highlights key aspects that are specific to studies on TTD: how participants were selected, the definition and measurement of TTD, and the analyses of the association between TTD and participant characteristics and health outcomes. The flowchart suggested to illustrate each step of the participant selection process could help raise awareness among authors, reviewers, and editors of threats to generalizability of study findings and conclusions. Indeed, the differences between the source and the analyzed populations were found to be an important source of concern in the abovementioned systematic review of studies on TTD [1].

A complete and accurate report of the definition and measurement of TTD is also of importance to enable readers to critically evaluate the risk that estimates of TTD may be biased by a subjective definition of time points (i.e., time of first symptoms and time of diagnosis). Whether the assessment of these time points was blinded to potential determinants and health outcomes is also critical.

Recommendations for the development of reporting guidelines suggest that key information concerning risk of bias should be included in all new or revised reporting guidelines. Ideally, identification of key information should rely on the empirical demonstration that it is related to risk of bias or threats to generalizability. For example, such demonstration has helped illustrate the influence of randomization methods on the estimation of a treatment effect in randomized controlled trials, supporting their emphasis in CONSORT [15]. In the present reporting guideline, judgment on each item’s potential influence on results was not based on an empirical demonstration, but on expert consensus. At present, an empirical demonstration is not possible, owing to the limited number of TTD studies investigating the same disease.

The methods used to develop this checklist are almost all in accordance with recommendations from the EQUATOR network, with a preparatory literature analysis [1] and vigilance about theoretical risk of bias and threats to generalizability [4]. For feasibility reasons, the discussion and refinement step was conducted by on-line discussions rather than a face-to-face meeting as recommended by the EQUATOR network. This on-line discussion could have limited the debate but it also allowed an independent expression of experts’ opinions without risk of authoritarian arguments. The response rate in the broad rating step was low (30 %), but the topics covered by the systematic review published by responding authors were representative of those of the invited authors. In the broad rating step, participants were all authors of systematic reviews; their methodological background is therefore likely to have been stronger than that of most potential users of our reporting guideline. An explanatory document was produced to help less initiated authors to properly use the reporting guideline.

## Conclusion

By estimating the magnitude of TTD for a disease, evaluating its potential consequences on prognosis and understanding its determinants, reports of studies on TTD may be used by clinicians and decision makers to plan corrective actions and identify inequities in access to healthcare. Critical appraisal is a key step before using results of clinical research for decisions in healthcare and policy. Incomplete and inaccurate reporting may prevent adequate critical appraisal of a study and lead to non-contributory scientific research [16]. Our reporting guideline provides authors, reviewers, and editors, as well as users of studies on TTD, with a tool that promotes complete and accurate reporting of studies.

## Additional files

Additional file 1: The 35-item checklist previously used to evaluate the completeness of reporting of studies on time to diagnosis. (DOCX 18 kb) Additional file 2: The 25-item checklist submitted to the scientific committee with a synthesis of the discussion and refinement steps (numbers of experts). Items in red were deleted after this first round. (DOCX 21 kb) Additional file 3: Distribution of the topics of the systematic reviews written (or co-written) by the invited and the responding authors in the broad rating step. (DOCX 17 kb) Additional file 4: Synthesis of ratings of the broad rating panel (numbers of experts). (DOCX 210 kb) Additional file 5: Explanatory document accompanying the checklist. (DOCX 19 kb)

## References

[1] Launay E, Morfouace M, Deneux-Tharaux C, Gras le-Guen C, Ravaud P, Chalumeau M (2014). Quality of reporting of studies evaluating time to diagnosis: a systematic review in paediatrics. Arch Dis Child.

[2] Brasme J-F, Morfouace M, Grill J, Martinot A, Amalberti R, Bons-Letouzey C (2012). Delays in diagnosis of paediatric cancers: a systematic review and comparison with expert testimony in lawsuits. Lancet Oncol.

[3] Scherdel P, Dunkel L, van Dommelen P, Goulet O, Salaün J-F, Brauner R (2016). Growth monitoring as an early detection tool: a systematic review. Lancet Diabetes Endocrinol.

[4] Launay E, Cohen JF, Morfouace M, Gras-Le Guen C, Ravaud P, Chalumeau M. Inadequate critical appraisal of studies in systematic reviews of time to diagnosis. J Clin Epidemiol. 2016. Ahead of print. doi:10.1016/j.jclinepi.2016.03.013. 10.1016/j.jclinepi.2016.03.013 27038853

[5] International Confederation of Childhood Cancer Parent Organizations. 2013. http://cms.onlinebase.nl/userfiles/c1icccpo/file/WHO_leaflet.pdf. Accessed 20 Sept 2016.

[6] National Library of Medicine. Early diagnosis. Medical Subject Headings. National Library of Medicine, Bethesda, USA. http://www.ncbi.nlm.nih.gov/mesh/?term=early+diagnosis. Accessed 28 Jun 2015.

[7] Brasme J-F, Chalumeau M, Oberlin O, Valteau-Couanet D, Gaspar N (2014). Time to diagnosis of Ewing tumors in children and adolescents is not associated with metastasis or survival: a prospective multicenter study of 436 patients. J Clin Oncol.

[8] Brasme J-F, Grill J, Doz F, Lacour B, Valteau-Couanet D, Gaillard S (2012). Long time to diagnosis of medulloblastoma in children is not associated with decreased survival or with worse neurological outcome. PLoS One.

[9] Moynihan R, Henry D, Moons KGM (2014). Using evidence to combat overdiagnosis and overtreatment: evaluating treatments, tests, and disease definitions in the time of too much. PLoS Med.

[10] von Elm E, Altman DG, Egger M, Pocock SJ, Gøtzsche PC, Vandenbroucke JP (2008). The Strengthening the Reporting of Observational Studies in Epidemiology (STROBE) statement: guidelines for reporting observational studies. J Clin Epidemiol.

[11] Bossuyt PM, Reitsma JB, Bruns DE, Gatsonis CA, Glasziou PP, Irwig L (2015). STARD 2015: an updated list of essential items for reporting diagnostic accuracy studies. BMJ.

[12] Weller D, Vedsted P, Rubin G, Walter FM, Emery J, Scott S (2012). The Aarhus statement: improving design and reporting of studies on early cancer diagnosis. Br J Cancer.

[13] Moher D, Schulz KF, Simera I, Altman DG (2010). Guidance for developers of health research reporting guidelines. PLoS Med.

[14] Development of a reporting guideline for reporting studies on time to diagnosis. http://www.equator-network.org/wp-content/uploads/2009/02/Reporting-studies-on-time-to-diagnosis-summary.pdf. Accessed 30 Aug 2015.

[15] Schulz KF, Chalmers I, Hayes RJ, Altman DG (1995). Empirical evidence of bias. Dimensions of methodological quality associated with estimates of treatment effects in controlled trials. JAMA.

[16] Glasziou P, Altman DG, Bossuyt P, Boutron I, Clarke M, Julious S (2014). Reducing waste from incomplete or unusable reports of biomedical research. Lancet.

