# Supplementary material for: Reporting studies on time to diagnosis: proposal of a guideline by an international panel (REST)
Source: BMC Med. 2016 Sep 27;14:146. doi: 10.1186/s12916-016-0690-7 (PMC5039933; doi:10.1186/s12916-016-0690-7)
Supplement: Additional file 1: — The 35-item checklist previously used to evaluate the completeness of reporting of studies on time to diagnosis. (DOCX 18 kb) [file 12916_2016_690_MOESM1_ESM.docx]

**Additional file 1**: 35-item checklist previously used to evaluate the completeness of reporting of studies on TTD^1^

| *Introduction* |
| --- |
| 1. State the aim of the study |
| 1. State frequency, morbidity and mortality of the studied condition |
| 1. State if there exists a treatment |
| 1. State if early diagnosis/treatment potentially changes the outcome |
| *Methods* |
| 1. Describe study population    1. Inclusion criteria    2. Exclusion criteria |
| 1. State if subgroups at risk of extreme time to diagnosis were excluded or analysed separately |
| 1. Describe how cases were identified |
| 1. List the symptoms allowing the physician to trigger diagnostic procedure (alert symptoms) during the study period |
| 1. State if all patients with alert symptoms underwent the reference diagnostic procedure |
| 1. Describe recruitment 2. Type (single centre, multicentre, population-based) 3. Locations 4. Setting (primary, secondary, or tertiary centre) 5. Period |
| 1. State the definition used for    1. T0 of illness (symptoms, signs)    2. Tx of diagnosis used in the study |
| 1. State the definition of the different time intervals within the time to diagnosis studied (patient interval, doctor interval) |
| 1. Describe    1. how many assessors evaluated time to diagnosis    2. if evaluations were independent    3. the degree of agreement about time to diagnosis between the different assessors    4. how potential disagreements were resolved |
| 1. Describe the qualification of the assessors who evaluated time to diagnosis |
| 1. State whether the persons who determined time to diagnosis were blinded to the outcome |
| 1. Definition of delayed diagnosis *(or in results)*    1. Defined with reference to literature    2. Arbitrarily defined    3. Defined as variation in the distribution of diagnostic delay observed in the study population |
| 1. Describe the statistical methods used to analyse the determinants and/or consequences of diagnostic delay |
| 1. Give a rationale for the sample size |
| 1. Describe how confounders were taken into account in the analysis of the determinants or consequences of diagnostic delay |
| *Results* |
| 1. Report number of 2. Eligible patients 3. Included patients |
| 1. Report the characteristics of the study population |
| 1. Report for the entire group 2. Mean/median diagnosis interval 3. Distribution of time to diagnosis |
| 1. Report the frequency of delayed diagnosis in the study population |
| 1. Report the determinants of diagnostic delay |
| 1. Report the consequences of diagnosis delay |
| *Discussion* |
| 1. Discuss key results and interpret them |
| 1. Compare results with those of previously published studies |
| 1. Discuss possible recruitment bias |
| 1. Discuss possible undiagnosed cases |
| 1. Discuss the bias in and the precision of the measurement of interval |
| 1. Discuss possible lead time bias |
| 1. Report if delayed diagnosis was too frequent or time to diagnosis too long |
| 1. Discuss avoidability of the delay |
| 1. Propose plan of action to reduce diagnostic delay |
| 1. Discuss the sensitivity, specificity and feasibility of this plan |
