# Supplementary material for: Reporting studies on time to diagnosis: proposal of a guideline by an international panel (REST)
Source: BMC Med. 2016 Sep 27;14:146. doi: 10.1186/s12916-016-0690-7 (PMC5039933; doi:10.1186/s12916-016-0690-7)
Supplement: Additional file 2: — The 25-item checklist submitted to the scientific committee with a synthesis of the discussion and refinement steps (numbers of experts). Items in red were deleted after this first round. (DOCX 21 kb) [file 12916_2016_690_MOESM2_ESM.docx]

**Additional file 2**: 25-item checklist submitted to the scientific committee with the synthesis of the discussion and refinement step (numbers of experts). Items in red were deleted after this first round.

| **Items** | **Is this item relevant? N=10*** | | **Is this item readable?**  **N=10*** | | |
| --- | --- | --- | --- | --- | --- |
|  | **Yes** | **No** | **Yes** | | **No** |
| 1. State whether subgroups at risk of extreme time to diagnosis were excluded or analysed separately | 9 | 1 | 9 | | 1 |
| 1. Describe how cases were identified | 10 | 0 | 5 | | 5 |
| 1. List the symptoms allowing the physician to trigger diagnostic procedure (alert symptoms) during the study period | 9 | 1 | 5 | | 5 |
| 1. State whether all patients with alert symptoms underwent the reference diagnostic procedure | 9 | 1 | 8 | | 2 |
| According to you, is there an item missing in the field of “identification of study population”? 3 experts answered yes  *- Whether a consecutive sample was included, Whether the study was prospective or retrospective (not sure if this should fall under Patients), Describe recruitment; setting, centres etc)*  *- Define period of study (patients collected from... To...), retrospective/prospective*  *- Yes—I would add: Was surveillance of symptoms and signs systematic or ad hoc?* | | | | | |
| 1. State the definition used for    1. T0 of illness (symptoms, signs)    2. Tx of diagnosis | 10 | 0 | 7 | | 3 |
| 1. #State the method used to estimate T0 and Tx (interviews, primary case-note audit, database analysis...) | 9 | 1 | 8 | | 2 |
| 1. #Give details on the method used to estimate T0 and Tx 2. For interviews: validation of the instrument used, timing from diagnosis, triangulation with other type of data 3. For primary case-note audit and database analysis: description of how information was extracted from case-note or database, description of database. | 8 | 2 | 8 | | 2 |
| 1. State the definition of the different time intervals within the time to diagnosis studied (patient interval, doctor interval) | 7 | 3 | 5 | | 5 |
| 1. Describe (for interviews and primary case note audit) 2. how many assessors evaluated time to diagnosis 3. if evaluations were independent 4. the degree of agreement about time to diagnosis between the different assessors 5. how potential disagreements were resolved | 9 | 1 | 9 | | 1 |
| 1. Describe the qualification of the assessors who evaluated time to diagnosis (for interviews and primary case note audit) | 9 | 1 | 10 | | 0 |
| 1. State whether the persons who determined and analysed time to diagnosis were blinded to the outcome | 7 | 3 | 8 | | 2 |
| 1. Definition of delayed diagnosis *(or in results)* 2. Defined with reference to literature 3. Arbitrarily defined 4. Defined as variation in the distribution of diagnostic delay observed in the study population | 9 | 1 | 8 | | 2 |
| According to you, is there an item missing in the field of “definition and measurement of time to diagnosis”? 2 experts answered yes  *- The OUTCOME should also be defined. That is not in the list, but pretty crucial I would think.*  *- Units? Days/hours/months/ years? What are the units for measuring time* | | | | | |
| 1. Describe how confounders were taken into account in the analysis of the determinants or consequences of diagnostic delay | 7 | 3 | 9 | | 1 |
| According to you, is there an item missing in the field of “determinants and consequences analyses”? 1 expert answered yes  *- Add another item: Describe the potential confounders?* | | | | | |
| 1. Report for the entire group 2. Mean/median diagnosis interval 3. Distribution of time to diagnosis | 10 | 0 | 9 | 1 | |
| 1. Report the frequency of delayed diagnosis in the study population | 7 | 3 | 9 | 1 | |
| 1. Report the consequences of delayed diagnosis | 6 | 4 | 7 | 3 | |
| According to you, is there an item missing in the field of “results”? 4 experts answered yes  *- Report outcome measures, like ORs or RRs*  *- Pathway has been important in my work previously; so, what are the components of “delay” – family, traditional healer, doctor, hospital, lab access*  *- I miss any reference and request for presenting standard errors or confidence intervals (precision parameters).*  *- I miss any item before #14 asking for description of study population (incl. subgroups) e.g. in a Table 1* | | | | | |
| 1. Discuss possible recruitment bias | 10 | 0 | 10 | 0 | |
| 1. Discuss possible undiagnosed cases | 9 | 1 | 9 | 1 | |
| 1. Discuss the bias in and the precision of the measurement of interval | 8 | 2 | 8 | 2 | |
| 1. Discuss possible lead time bias | 10 | 0 | 10 | 0 | |
| According to you, is there an item missing in the field of “discussion of study limitations”? 2 experts  *- Yes—discuss possible bias due to non-blinding.*  *- If disease doesn’t present symptomatically, diagnosis secondary to something else can be complicated* | | | | | |
| 1. Discuss possible lead time bias. | 10 | 0 | 10 | 0 | |
| 1. Report if delayed diagnosis was too frequent or time to diagnosis too long | 3 | 7 | 3 | 0 | |
| 1. Discuss avoidability of delayed diagnosis | 6 | 4 | 8 | 2 | |
| 1. Propose plan of action to avoid delayed diagnosis delay | 4 | 6 | 3 | 0 | |
| 1. Discuss the sensitivity, specificity and feasibility of this plan | 2 | 8 | 1 | 6 | |
| According to you, is there an item missing in the field of “discussion of clinical application”? 2 experts answered yes  *- Yes—priorities for future studies*  *- Discuss subcomponents/pathways of delay* | | | | | |

* One expert gave a general feedback on the checklist but did not rate each item

# Items common with the Aarhus statement
