# Supplementary material for: Reporting studies on time to diagnosis: proposal of a guideline by an international panel (REST)
Source: BMC Med. 2016 Sep 27;14:146. doi: 10.1186/s12916-016-0690-7 (PMC5039933; doi:10.1186/s12916-016-0690-7)
Supplement: Additional file 3: — Distribution of the topics of the systematic reviews written (or co-written) by the invited and the responding authors in the broad rating step. (DOCX 17 kb) [file 12916_2016_690_MOESM3_ESM.docx]

**Additional file 3:** Distribution of the topics of the systematic reviews written (or co-written) by the invited and responding authors in the broad rating step.

| **Systematic Review Topic** | **Invited authors (N=98)** | **Respondents (N=29)** |
| --- | --- | --- |
| Cancer | 60 (61%) | 15 (52%) |
| Tuberculosis | 18 (18%) | 4 (14%) |
| Stroke | 6 (6%) | 2 (7%) |
| Arthritis | 4 (4%) | 3 (10%) |
| Dementia, narcolepsy, HIV infection, somatoform disorders | 2 (2% each) | 1 (3% each) |
| Myocardial infarction | 1 (1%) | 1 (3%) |
| Inflammatory bowel disease | 1 (1%) | 0 |
