# Supplementary material for: Reporting studies on time to diagnosis: proposal of a guideline by an international panel (REST)
Source: BMC Med. 2016 Sep 27;14:146. doi: 10.1186/s12916-016-0690-7 (PMC5039933; doi:10.1186/s12916-016-0690-7)

**Additional file 4:** Explanatory document accompanying the checklist.

Notice:

Here, authors, reviewers and editors of reports of studies on TTD will find explanations on and/or justifications of some items of the checklist. Most items are illustrated with examples extracted from 4 published reports of studies on TTD.

- Article 1, reports a study which aimed to analyse TTD of medulloblastoma in children and its association with patients’ outcomes.^8^
- Article 2, reports a study which aimed to analyse TTD in imported malaria and the frequency of delayed diagnosis.^17^
- Article 3, reports a study which aimed to quantify delayed diagnosis in retinoblastoma and to study its association with patients’ outcomes.^18^
- Article 4, reports a study which aimed to analyse TTD of medulloblastoma in children and its potential determinants.^19^

Items marked with a cross « †» are common with other reporting guidelines (CONSORT, STARD, STROBE) in their meaning, items marked with an asterisk « * » are optional depending on the study objectives.

**ITEM 1 (Title). Identify the article as a study on time to diagnosis.**

**Example (article 1)**

“*Long time to diagnosis of medulloblastoma in children is not associated with decreased survival or with worse neurological outcome*”

Time to diagnosis is defined by the interval of time between the first symptom of a disease and the time of the diagnosis. Identifying the article as a study on time to diagnosis may ease the retrieval of the study in electronic databases (for the need of a systematic review, for example). There are many terms associated with the concept of time to diagnosis, such as “time to diagnosis”, “delay”, “pre-diagnostic symptomatic interval”, “symptom interval” or “lag time". The most frequent keywords (MeSH heading) in the National Library of Medicine’s MEDLINE database associated with 95 studies on time to diagnosis included in a previous systematic review on time to diagnosis in pediatrics and in a systematic assessment of systematic reviews on time to diagnosis were: “time factors” (introduced in 1999) (n=58), “delayed diagnosis” (introduced in 2010) (n=25) and “early diagnosis” (introduced in 2004) (n=17). None of these keywords had enough sensitivity to identify all studies and “time factors” is poorly specific (more than 1 million hits in PubMed in January 2015). We recommend the use of “time to diagnosis” to express the time interval between the first symptoms and the diagnosis, and the term “delayed diagnosis” to refer to an abnormally long time to diagnosis.

**ITEM 2A (Introduction section). Explain the scientific background and rationale for the study.**

**Example (article 1)**

*“Brain tumors are the* ***leading cause of solid cancers*** *in children [1]. Medulloblastoma, one of the most common types [1], has a* ***10-year survival rate of 50% [2–5], and many survivors have neurological and cognitive sequelae*** *[6,7].* ***The time to diagnosis for brain tumors is one of the longest of all childhood cancers****, with a median ranging from 2 to 5 months [8–24](…)* ***The delay in diagnosis of childhood tumors leads to painful remorse or guilt feelings*** *for parents and physicians, loss of confidence and sometimes conflicts [26,27]. (…) Only two studies are available for medulloblastoma specifically: one found no relation in either direction between delay and survival [17], while the other reported an inverse relation between duration of symptoms and metastasis [13]. Nonetheless* ***several factors limit the usefulness of these results****: the limited numbers of pediatric patients [13,17], pooled analyses for pediatric and adult patients [13], single-center recruitment subject to selection bias [13], incomplete initial disease staging for some patients that increases the likelihood of classification bias [13], a study period partially preceding the availability of CT and MRI [13], and a lack of multivariate analyses despite the presence of potential confounders [13,17]. Finally, none of these studies analyzed the relation between time to diagnosis and either local tumor stage, complete surgical resection or neurological and cognitive sequelae.”*

In the introduction section, authors should report key elements of the scientific background, including the frequency, the morbidity and the mortality of the studied condition. They should also precise if a treatment exists and if there are data in the literature to support the benefit of early diagnosis and treatment. They should should summarize the body of evidence concerning TTD, its potential determinants and/or its potential consequences on health outcome (depending on the aim of the study), underline gaps in this knowledge, if any, and justify what their study is intended to add.

**ITEM 2B (Introduction section). State specific objective(s).**

**Example (article 1)**

*“****Our objective*** *was therefore to analyze, in a pediatric population-based study****, the consequences of the time to diagnosis of medulloblastoma on initial tumor stage, survival, and neuropsychological and neurological outcome****, while taking confounding factors into account”*

The objectives of a study on time to diagnosis may be non-exclusively to assess a time to diagnosis or its change over time, to compare times to diagnosis or to~~o~~ study the association between time to diagnosis and other variables such as participant characteristics (e.g., socio-demographic status, age…), healthcare characteristics (level of care, presence of specialists of the disease condition, location...) or health outcomes such as survival. The design of the study and the items to be reported will depend on this (these) objective(s).

**ITEM 3. (Methods section) Describe the setting, location(s), and relevant dates, including periods of recruitment.**

**Example (article 1)**

*“We conducted a* ***multicenter historic population-based cohort*** *study that included* ***all patients in one French region*** *(Ile-de-France, the Paris metropolitan region) (…)* ***from 1990 through 2005****.”*

As time to diagnosis may vary with settings and location and may change over time, it is important to report these elements in the methods section. One could hypothesize, for example, that time to diagnosis may be shorter or longer in a reference centre compared to less specialised settings. Moreover, time to diagnosis may vary within the year and be shorter when diagnosis is more frequent or can change over time after interventions. Time to diagnosis may also vary geographically according to medical environment (physicians’ density for example).

**ITEM 4 (Methods section). State eligibility criteria of participants (i.e., inclusion and exclusion criteria, especially diagnostic criteria).**

**Example (article 1)**

“(…) *that included all patients in one French region (Ile-de- France, the Paris metropolitan region) who* ***were younger than 15 years*** *when diagnosed with a histologically-confirmed medulloblastoma. (…).* ***Pathologists at each hospital (sites of the neurosurgery and oncology departments) confirmed all histological diagnoses of biopsy or resection samples within days of the radiological diagnosis****.”*

Studies on time to diagnosis focus on patients with a positive diagnosis. Authors must detail the diagnosis criteria used in order to help readers to evaluate whether they can be considered accurate.

**ITEM 5 (Methods section).** **Describe the source population [i.e., the population with signs and symptoms that usually trigger healthcare professionals to initiate the diagnostic procedure(s)] and how the participants were identified within it.**

**Example (article 2)**

*“In the participating emergency departments, the* ***routine protocol*** *for* ***children with fever was to ask if they had recently visited a malaria-endemic area****. If so, the search for Plasmodium trophozoites in* ***thick and/or thin blood smears*** *was ordered.”*

Here authors should describe the population with signs and symptoms that are usually used by healthcare professionals to trigger the diagnostic procedure (in this example: children with fever and having recently visited a malaria-endemic area). They should also state whether the surveillance of these signs and symptoms was performed routinely or *ad hoc* (in this example, authors stated that all the children with fever and having visited an endemic area were routinely tested for malaria during the study period). They should also state the mechanism for identifying participants among the source population and how participants were sampled (consecutive or random sampling, other).

**ITEM 6 (Methods section). *State how known subgroups of participants with an inherently short or long time to diagnosis were handled (e.g., by subgroup analysis, exclusion).**

**Example (article 3)**

*“The clinical charts of 64 consecutive patients who presented to the Memorial Sloan-Kettering Cancer Center with newly diagnosed retinoblastoma between November 11, 1993, and January 14, 1998, were reviewed. Seven* ***patients with a family history of retinoblastoma were excluded*** *from the results.”*

Some participants may have intrinsic characteristics associated with a risk of longer or shorter time to diagnosis. In this example, potential participants with known family history of retinoblastoma, who are at risk of shorter time to diagnosis compared with cases without family history of retinoblastoma, were excluded.

**ITEM 7 (Methods section).** **Define time points (e.g., time of first signs and symptoms, time of diagnosis) and *time intervals (e.g., patient or physician intervals).**

**Example (article 1)**

*“The* ***time to diagnosis, expressed in days, was defined as the interval between the first symptom attributable to the disease and the date of diagnosis (date of brain imaging)****.*

**Example (article 2)**

*“****Patient delay*** *was defined as the* ***time between fever onset and the first medical consultation****.* ***Doctor delay*** *was the* ***time between the first consultation and the parasitological diagnosis****. The* ***total delay*** *was the sum of the two. We defined* ***a late medical diagnosis*** *as a doctor delay of* ***longer than 12 h****”*

Authors should detail how they defined time points in order to help readers evaluate whether they are accurate. When there are several signs and symptoms attributable to the studied condition (as in Article 2), authors should list all of them. Sub-divisions composing time to diagnosis should also be defined depending on the objective of the study. Subdividing time to diagnosis may help target corrective actions (health education and/or medical education) by measuring the role of each participant in the delay (patients/parents, physicians/healthcare system). The use of a theoretical framework may be useful to report these subdivisions. Example of the complete framework suggested in the Aarhus statement ^20^. Such framework can be customised to fit any study on time to diagnosis.


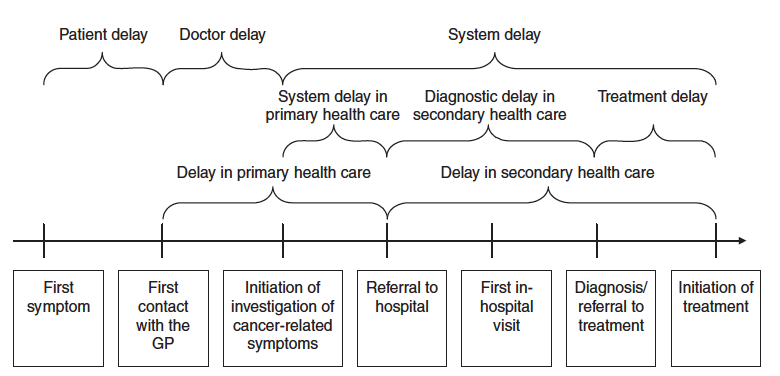


**ITEM 8 (Methods section). State the methods used to collect study data (e.g., extraction from medical records, participant interview or questionnaires, analysis of an already existing database, other)**.

**Example (article 1)**

*“****We collected the following data from each medical file*** *in the neurosurgery and oncology departments (…)”*

Authors should report how data were collected (i.e., extraction from medical records, participant interview or questionnaires, analysis of an already existing database, other) Authors should also state whether the data were collected prospectively or retrospectively. Finally, authors should report whether they performed a triangulation with other types of data to enhance the validity of collected data.

**ITEM 9 (Methods section). Describe how time points were assessed (e.g., number of assessors, their qualifications)**.

**Example (article 1)**

*“The time to diagnosis, expressed in days, was defined as the interval between the first symptom attributable to the disease and the date of diagnosis (date of brain imaging).* ***When ambiguous (for 2% of patients), it was independently evaluated by 3 of the authors, who reached a consensus.****”*

Time points may be subjective. For example, when the first signs and symptoms are not specific of the studied condition or when the diagnosis criteria are not obvious. Authors should also report whether time points could be easily retrieved (e.g., whether the precise dates of first symptoms and diagnosis are available in a database) and whether they were potentially ambiguous. If the assessment of time points is potentially ambiguous (e.g., retrospective assessment from medical charts), authors should report how many assessors collected the time points, with their qualifications, whether they were independent, and how any disagreements were resolved.

**ITEM 10A (Methods section). *If the study aims to evaluate associations between participant characteristics and time to diagnosis, state whether assessors of time to diagnosis were blinded to these characteristics.**

Participant characteristics is a broad term that can include sociodemographic characteristics, disease characteristics (e.g., tumor stage, severity) or even healthcare system characteristics such as the level of care and the specialty of the physician in charge. Authors should state whether assessors of time to diagnosis were blinded to these characteristics in order to help readers evaluate the risk of measurement bias inherent in the lack of blinding: time to diagnosis may be systematically over- or underestimated when some characteristics are present and known to the assessor of time points.

**ITEM 10B (Methods section). *If the study aims to evaluate associations between time to diagnosis and participant health outcomes (e.g., survival), state whether assessors of time to diagnosis were blinded to these outcomes.**

Authors should state whether assessors of time to diagnosis were blinded to health outcomes (survival, sequelae, …) because of the risk of measurement bias inherent to the lack of blinding: time to diagnosis may be systematically over- or underestimated if the assessor is aware of a given health outcome.

**ITEM 11 (Methods section). Describe the statistical methods used, including whether time to diagnosis was analysed as a continuous or categorized variable (e.g., delayed versus not delayed).**

**Example (article 1):**

*“The time to diagnosis was used as a binary variable after dichotomization around the median or as a continuous variable (after testing linearity). Age was dichotomized around 5 years, in view of the difference in treatment around this age.”*

Time to diagnosis is a quantitative variable; it can be analysed as a linear variable or transformed (e.g., in polynoma, logarithm) or categorized (as in the example).

**ITEM 12 (Methods section). *If the study aims to evaluate associations between time to diagnosis and other factors (e.g., participant characteristics or health outcomes), describe which confounders were considered and how they were chosen, measured and analysed.**

**Example (article 1)**

*“Second, we* ***studied the relation between this prediagnosis interval and initial severity*** ***factors*** *(****metastasis, tumor volume, T stage and completeness of resection****) by univariate (Kruskal-Wallis test) and* ***multivariate analysis****, taking into consideration* ***the cofactors of interest****, by either* ***stratification or adjustment*** *in a* ***logistic regression*** *model.”*

Authors should justify their choice of potential confounders and state whether this choice was made *a priori*. Authors should define how they measured and analyzed potential confounders (as continuous linear, transformed or categorical variables).

**ITEM 13 (Methods section). †Give a rationale for the sample size.**

The sample size conditions the precision of the estimates of time to diagnosis. The sample size is also important when studying associations between time to diagnosis and other factors (health outcomes or patients characteristics) to evaluate if it is large enough to bring out these associations (power).

**ITEM 14 (Results section). Report the number of individuals at each step of the selection process between the source population and participants with data analysed, and provide a flowchart (see example). Give reasons for non-participation at each stage.**

**Suggestion of flowchart** (See Figure 2)

Authors are not expected to provide all numbers (N) because some may be unknown, but authors should identify each step of the selection process in order to help readers interpret the results. This flowchart follows the steps of population selection as defined in items 4 and 5.

**ITEM 15 (Results section). †Report demographic and clinical characteristics of participants.**

**Example (article 1**)

*“Median age at the first symptom was 6 years (interquartile range (IQR): 4–9) and 31% were younger than 5 years. The patients had a local tumor in 62% (standard risk 40%, local high risk 22%) and a metastatic tumor in 38% of cases. The median tumor volume was 33 cm3 (IQR 22–42). Staging showed 2% of the children had T1 local tumors, 27% T2, 15% T3A, 39% T3B, and 17% T4…”*

Authors should give the description of the demographic and clinical characteristics of participants so that readers can evaluate the generalizability of the results. This description may be given in a table.

**ITEM 16 (Results section). Report the distribution of time to diagnosis.**

**Example (article 1)**

*“Time to diagnosis was determined in all cases. Its median was 65 days (IQR 31–121, range 3–457).”*

Authors should report the distribution of time to diagnosis so that readers can evaluate the existence of extreme time to diagnosis. Time to diagnosis is a left-censored quantitative variable that should be reported with median and interquartile range and/or extreme.

**ITEM 17 (Results section). *If associations between time to diagnosis and other factors (e.g., participant characteristics or health outcomes) are described, report measures of association and their precision (e.g., confidence intervals).**


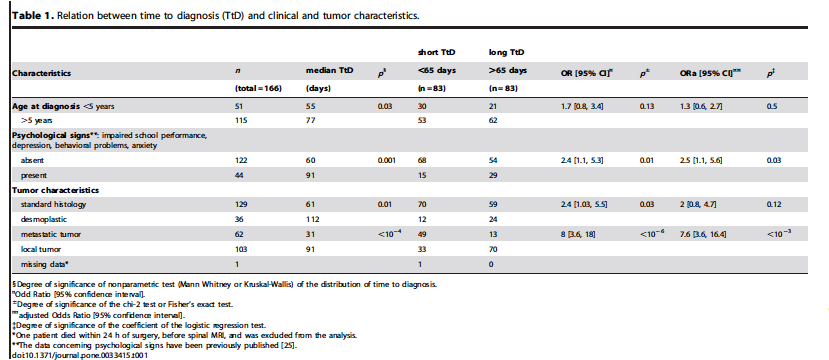
**Example (article 1)**

If associations between time to diagnosis and other factors are assessed, authors should report measures of associations and their precision so that the reader could evaluate their magnitude and the degree of uncertainty around estimates.

**ITEM 18 (Discussion section). †Summarize key results with reference to study objectives and discuss their potential clinical implications.**

**Example (article 1)**

*“We found complex and often inverse relations between a longer time to diagnosis of medulloblastoma in children, the initial severity factors, and survival. A long prediagnosis interval was associated with a larger tumor volume, a lower frequency of metastasis, desmoplastic histology, and longer survival in the univariate analysis but not after adjustment. The time to diagnosis was significantly associated with IQ score among survivors. No significant relation was found between the time to diagnosis and neurological disability. In the 62 patients with metastatic disease, a long prediagnosis interval was associated with a more advanced T stage, fourth ventricle floor invasion, and incomplete surgical resection; it nonetheless did not influence survival significantly in this subgroup.”*

**Example (article 4)**

*“Is it possible to reduce the diagnosis delay of medulloblastoma in children? The indications for brain imaging could be expanded to the children with nonspecific symptom that persist for more than a month, or a combination, even nonspecific, of symptoms, or a significant neurological sign or symptom (such as ataxia), as suggested by the Children's Brain Tumour Research Centre [41], even if the specificity of such a strategy would probably be low, given the frequency of these symptoms in the general population [1, 3]”*

Authors are expected to discuss their main results and their clinical implications (for example whether and how TTD could be reduced).

**ITEM 19A (Discussion section). Discuss sources of potential bias, including bias due to the selection of participants from the source population (e.g., undiagnosed cases) and to the assessment of time points.**

**Example (article 1)**

*“The principal limitation of the study is its retrospective nature. Nonetheless the data came from multiple sources and the time to diagnosis was ambiguous for only 2% of patients. Disease extension was determined in all but one patient (<1%), based on standardized measurement methods (imaging). No patients were lost to follow-up.”*

**Example (article 4)**

*“This exhaustiveness was verified for a 5-year period by the French National Paediatric Cancer Registry. As in any disease, the possibility of death before diagnosis cannot be ruled out.”*

Because studies on time to diagnosis are based only on diagnosed cases, authors should be encouraged to discuss the extent to which undiagnosed cases could have affected results, notably with help of the proposed flowchart. When time points were potentially ambiguous, authors should discuss the impact of a potential classification bias.

**ITEM 19B (Discussion section). *If association between time to diagnosis and survival was studied, discuss possible lead-time bias.**

In case of lead-time bias, some could falsely conclude to longer perceived survival time 2 than perceived survival time 1.


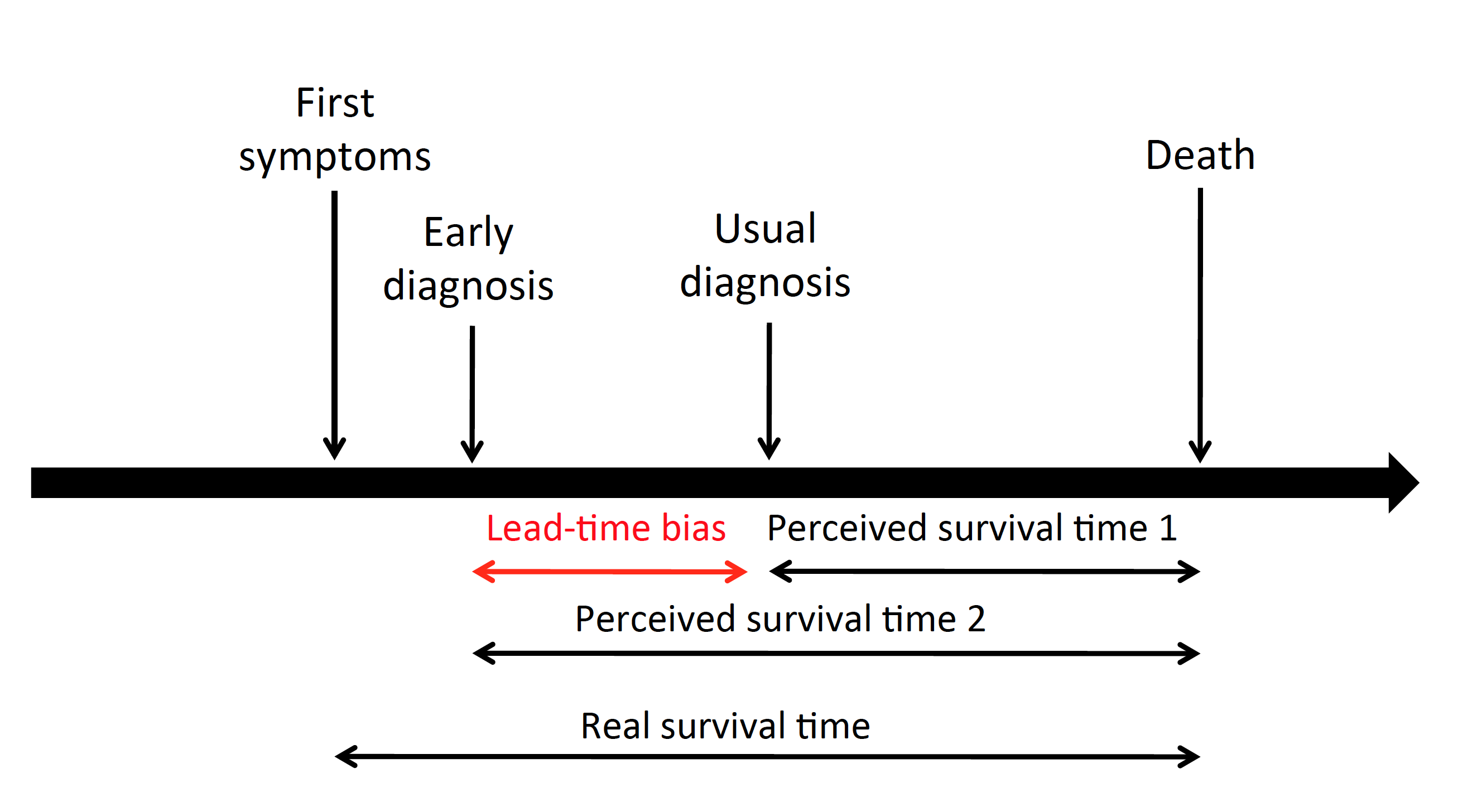

Supplement: Additional file 4: — Synthesis of ratings of the broad rating panel (numbers of experts). (DOCX 210 kb) [file 12916_2016_690_MOESM4_ESM.docx]
