# Supplementary material for: Reporting studies on time to diagnosis: proposal of a guideline by an international panel (REST)
Source: BMC Med. 2016 Sep 27;14:146. doi: 10.1186/s12916-016-0690-7 (PMC5039933; doi:10.1186/s12916-016-0690-7)
Supplement: Additional file 5: — Explanatory document accompanying the checklist. (DOCX 19 kb) [file 12916_2016_690_MOESM5_ESM.docx]

**Additional file 5:** Synthesis of ratings of the broad rating panel (numbers of experts)

| **Items** | Relevant | | | Readable | |
| --- | --- | --- | --- | --- | --- |
|  | **Yes** | **No** | **I don’t know** | **Yes** | **No** |
| 1. Identify the article as a study on time to diagnosis | 25 | 1 | 3 | 22 | 4 |
| 2a. †Explain the scientific background and rationale for the study | 25 | 0 | 1 | 26 | 0 |
| 2b. State specific objective(s) | 24 | 1 | 1 | 25 | 1 |
| 3. Describe the setting, location(s), and relevant dates, including periods of recruitment | 25 | 0 | 1 | 24 | 2 |
| 4. State eligibility criteria of participants (i.e., inclusion and exclusion criteria, especially diagnostic criteria) | 25 | 0 | 1 | 24 | 2 |
| 5. Describe the source population [i.e., the population with signs and symptoms that usually trigger healthcare professionals to initiate the diagnostic procedure(s)] and how the participants were identified within it | 24 | 0 | 1 | 20 | 3 |
| 6. State how known subgroups of participants with an inherently short or long time to diagnosis were handled (e.g., by subgroup analysis, exclusion) | 22 | 1 | 1 | 17 | 5 |
| 7. Define time points (e.g., time of first signs and symptoms, time of diagnosis) and time intervals (e.g., patient or physician intervals) | 24 | 0 | 0 | 23 | 0 |
| 8. †State the methods used to collect study data | 23 | 0 | 1 | 23 | 0 |
| 9. Describe how time points were assessed (e.g., number of assessors, their qualifications) | 23 | 1 | 0 | 21 | 2 |
| 10a. *If the study aims to evaluate associations between participant characteristics and time to diagnosis, state whether assessors of time to diagnosis were blinded to these characteristics | 22 | 2 | 0 | 21 | 2 |
| 10b. *If the study aims to evaluate associations between time to diagnosis and participant health outcomes (e.g., survival), state whether assessors of time to diagnosis were blinded to these outcomes | 22 | 2 | 0 | 22 | 1 |
| 11. Describe the statistical methods used, including whether time to diagnosis was analysed as a continuous or categorized variable (e.g., delayed versus not delayed) | 24 | 0 | 0 | 22 | 1 |
| 12. *If the study aims to evaluate associations between time to diagnosis and other factors (e.g., participant characteristics or health outcomes), describe which confounders were considered and how they were chosen, measured and analysed | 22 | 1 | 1 | 19 | 4 |
| 13. †Give a rationale for the sample size | 21 | 0 | 3 | 22 | 1 |
| 14. Report the number of individuals at each step of the selection process between the source population and participants and provide a flowchart (see example). Give reasons for non-participation at each stage | 21 | 1 | 2 | 20 | 3 |
| 15. †Report demographic and clinical characteristics of participants | 24 | 0 | 0 | 23 | 0 |
| 16. Report the distribution of time to diagnosis | 24 | 0 | 0 | 23 | 0 |
| 17. *If associations between time to diagnosis and other factors (e.g., participant characteristics or health outcomes) are described, report measures of association and their precision (e.g., confidence intervals) | 23 | 1 | 0 | 13 | 1 |
| 18. †Summarize key results with reference to study objectives and discuss their potential clinical implications | 24 | 0 | 0 | 13 | 1 |
| 19a. Discuss sources of potential bias, including bias due to the selection of participants from the source population (e.g., undiagnosed cases) and to the assessment of time points | 24 | 0 | 0 | 14 | 0 |
| 19b. *If association between time to diagnosis and survival was studied, discuss possible lead-time bias | 21 | 0 | 3 | 12 | 2 |

†Items common with other reporting guidelines (CONSORT, STARD, STROBE) in their meaning

*Optional items depending on the studied condition and/or the study objectives.
